# Association between malocclusion and temporomandibular joint disorders: evidence from a Mendelian randomization study

**DOI:** 10.22514/jofph.2026.062

**Published:** 2026-09-12

**Authors:** Zhouqiang Wu, Sen Wang, Chang Liu, Zhiyi Zhao, Yan Feng, Yue Shan, Wen Li, Mengjie Wu

**Affiliations:** ^1^Department of Orthodontics, Stomatology Hospital, School of Stomatology, Zhejiang University School of Medicine & Clinical Research Center for Oral Diseases of Zhejiang Province & Key Laboratory of Oral Biomedical Research of Zhejiang Province & Cancer Center of Zhejiang University, 310006 Hangzhou, Zhejiang, China; ^2^Department of Orthodontics, State Key Laboratory of Oral Diseases, West China Hospital of Stomatology, Sichuan University, 610000 Chengdu, Sichuan, China

**Keywords:** Malocclusion, Temporomandibular joint disorders, Mendelian randomization

## Abstract

**Background**: Although malocclusion and temporomandibular joint disorders (TMD) frequently co-occur in clinical practice, controversy persists regarding whether this overlap reflects a pathophysiological causal relationship or incidental concurrence. This study aimed to investigate the potential association between malocclusion and TMD using a bidirectional two-sample Mendelian randomization (MR) approach. **Methods**: Bidirectional two-sample MR was performed to evaluate the potential causal relationship between malocclusion and TMD. Summary statistics for malocclusion were obtained from the FinnGen consortium, while TMD datasets were obtained from the FinnGen consortium and an independent genome-wide association study (GWAS) of the UK Biobank (UKB). To account for multiple comparisons, a Bonferroni-corrected significance threshold of 0.00625 was applied. The strength of the instrumental variables (IVs) was assessed using *F*-statistics. Causal inference was conducted using multiple MR methods, including inverse variance weighted (IVW), maximum likelihood estimation, MR-Egger regression, weighted median (WM), and penalized weighted median analyses. Sensitivity analyses were performed using Cochran’s Q test for heterogeneity, MR-Egger intercept analysis, Mendelian Randomization Pleiotropy RESidual Sum and Outlier (MR-PRESSO) global outlier assessment, and iterative leave-one-out validation. **Results**: Convergent evidence from IVW, WM, and penalized weighted median estimators suggested potential causal effects of distal bite, deep bite, and open bite on TMD susceptibility. After Bonferroni correction, deep bite remained significantly associated with TMD. The mean *F*-statistics for the IVs ranged from 22.23 to 23.62 across malocclusion types, indicating adequate instrument strength. Reverse MR analysis using 130 TMD-related Single Nucleotide Polymorphisms (SNPs) showed no evidence supporting a causal effect of TMD on malocclusion. **Conclusions**: This MR study provides evidence that several malocclusion types, including distal bite, deep bite, and open bite, are potentially causally associated with TMD, with deep bite showing the strongest association.

## 1. Introduction

Temporomandibular joint disorders (TMD) represent a clinically heterogeneous category of musculoskeletal conditions that compromise the function of the jaw apparatus and frequently lead to chronic orofacial pain. Under the widely adopted Diagnostic Criteria for Temporomandibular Disorders (DC/TMD) framework, this diagnostic umbrella encompasses several pathologically distinct entities, including inflammatory and degenerative joint processes, myofascial pain syndromes, internal derangements characterized by articular disc displacement, and joint laxity conditions [1]. Patients affected by these disorders typically present with symptoms such as limitation of jaw movement, periauricular discomfort, recurrent headache, and facial pain. From an epidemiological perspective, the burden of TMD differs markedly across life stages, with adult and geriatric populations experiencing symptoms at substantially higher frequencies, reported at nearly one in three, whereas pediatric and adolescent groups show considerably lower occurrence rates, at approximately one in nine [2]. The etiology of TMD is multifactorial and involves biological (*e.g.*, malocclusion), psychological, environmental, and social factors [3].

Malocclusion refers to deviation from normal dental alignment or an improper occlusal relationship between the maxillary and mandibular dentition. This common dental anomaly can considerably reduce an individual’s quality of life. Malocclusion encompasses both dental misalignment and skeletal discrepancies involving the jaw bones, and both forms may contribute to TMD. Various forms of malocclusion have been described, including distal bite, mesial bite, crossbite, scissors bite, deep bite, and open bite. Malocclusion is primarily caused by genetic and environmental factors that influence the development of the jaws and teeth.

Previous studies have reported that individuals with malocclusion frequently experience TMD-related symptoms. Individuals presenting with a distal bite, for instance, tend to be associated with joint-related TMD, whereas hypodivergent/deep-bite-prone exhibit bilaterally increased activity in the masseter muscles during mastication [4, 5]. Similarly, posterior crossbite has been linked to unequal muscular function and modified condylar positioning, both of which may precipitate cumulative joint strain over time [6]. One possible explanatory mechanism involves the aberrant mechanical forces transmitted to the temporomandibular joint and its periarticular tissues as a result of deviant dental alignment. By disrupting muscular equilibrium in the jaw, malocclusion may require the masticatory muscles to generate additional effort to sustain functional performance. This compensatory overexertion may subsequently contribute to pain and inflammatory changes within the temporomandibular joint [7, 8]. Additionally, condylar asymmetry, which frequently co-occurs with malocclusion, particularly unilateral posterior crossbite, may alter the distribution of mechanical forces across the temporomandibular joint, contributing to degenerative changes and TMD-related symptoms [9, 10].

Beyond its effects on musculature and condylar morphology, malocclusion can also influence the positioning and kinematics of the temporomandibular joint disk, which is a fibrocartilaginous interpositional structure situated between the mandibular condyle and the temporal bone [11]. Abnormal disk–condyle spatial relationships or deranged disk movement can instigate compression neuropathies and capsular inflammatory reactions, ultimately leading to the characteristic symptoms of mechanical joint dysfunction [11]. Taken together, these converging lines of evidence indicate that malocclusion may be an important etiological contributor and disease-aggravating factor within the TMD pathological continuum.

However, the exact nature of the malocclusion–TMD relationship has yet to be fully elucidated. While observational investigations have documented a correlation between the two conditions, ascertaining whether malocclusion constitutes a causative agent or an outcome of TMD remains methodologically challenging. Moreover, confounding factors, such as genetic predispositions and lifestyle habits, may further complicate the interpretation of these findings.

The association between malocclusion and TMD represents an important research area with the potential to improve our understanding of the etiology and treatment of TMD. The primary objective of this study was to evaluate whether genetically determined susceptibility to distinct categories of malocclusion confers an elevated risk for TMD and, reciprocally, whether genetic predisposition to TMD raises the likelihood of malocclusion, thereby clarifying the causal direction underlying this association. To address these questions, we employed Mendelian randomization (MR) analyses to investigate the potential causal links between various types of malocclusion and TMD.

## 2. Methods

### 2.1 Study design

A bidirectional MR design (Fig. 1) was employed to evaluate the causal effect of exposure on the outcome and *vice versa*. This study explored the potential relationships between various types of malocclusion, including distal bite (Class II malocclusion), mesial bite (Class III malocclusion), crossbite, scissor bite, deep bite, open bite, deep bite surgery, and open bite surgery, and temporomandibular disorders (TMD) using bidirectional two-sample MR analysis, based on summary statistics from genome-wide association studies (GWAS).

**Fig. 1.** Schematic overview of the study design. IVs: instrumental variables; TMD: temporomandibular joint disorders; MR: Mendelian randomization; IVW: inverse variance weighted; SNPs: Single Nucleotide Polymorphisms.

### 2.2 Data source

Malocclusion-related GWAS summary statistics were retrieved from the FinnGen consortium, and the numbers of cases and controls are listed in Table 1.

**Table 1.** GWAS data sources for different types of malocclusion.

Phenotype	No. of cases	No. of controls
Mesial bite	254	411,927
Crossbite	1952	410,229
Scissor bite	625	411,556
Open bite that required surgery (Lefort1 or BSSRO)	268	411,913
Open bite	1449	410,732
Distal bite	946	411,235
Deep bite that required surgery	336	411,845
Deep bite	3338	408,843

*BSSRO: bilateral sagittal split ramus osteotomy.*

TMD data were also sourced from the FinnGen consortium using the phenocode “TEMPOROMANDIB” (https://r10.finngen.fi/pheno/TEMPOROMANDIB) during the discovery phase, comprising 6314 affected individuals and 222,498 unaffected comparators. Replication data from the UK Biobank (UKB) GWAS included 217 TMD cases and 456,131 controls of exclusively European genetic background [12]. Both the FinnGen and UKB datasets were publicly available summary statistics, and no individual-level participant data were accessible. Consequently, specific TMD subclassifications according to the DC/TMD criteria could not be distinguished in this analysis.

To satisfy the first MR assumption and obtain a sufficient number of single-nucleotide polymorphisms (SNPs), instrumental variables (IVs) strongly associated with malocclusion and TMD were selected using a significance threshold of *p* < 5 × 10^−6^. SNPs in strong linkage disequilibrium (LD) were subsequently excluded using a threshold of *r*^2^ < 0.01 within a 5000-kb window. To satisfy the third MR assumption, namely no direct effect of IVs on the outcome, SNPs associated with the outcome at *p* < 5 × 10^−8^ were excluded.

Statistical power was calculated using the formula for the inverse variance weighted (IVW) estimator. The variance of the causal effect estimate was derived from the harmonized SNP-level data as Var(β_IVW_) = 1/Σ(w_i_), where w_i_ = (β_exposure,i_)^2^/(SE_outcome,i_)^2^. The minimum detectable odds ratio (MDOR) at 80% power was calculated as MDOR = exp[(Z_0.80_ + Z_1 − α/2_) × SE(β_IVW_)]. Power to detect a clinically meaningful Odds Ratio (OR) of 1.1 was computed at both α = 0.05 and the Bonferroni-corrected α = 0.00625. The proportion of variance in exposure explained by IVs (*R*^2^) was estimated from SNP-level effect estimates as *R*^2^ = Σ(2β^2^MAF(1 − MAF))/(1 + Σ(2β^2^MAF(1 − MAF))), with LD adjustment.

Finally, the palindromic and incompatible SNPs were excluded by harmonizing the exposure and outcome data. Harmonization involved three sequential quality-control steps: (a) effect allele alignment to the same reference strand across FinnGen and UK Biobank datasets, after which effect alleles were fully concordant, with 0 allele mismatches across all 582 harmonized SNPs; (b) exclusion of palindromic SNPs (Adenine/Thymine (A/T) or Guanine/Cytosine (G/C)) with minor allele frequencies between 0.42 and 0.58, whereby a total of 47 palindromic SNPs were identified, all with Expected average frequency (EAF) below 0.42 (range: 0.000–0.203), and were retained after strand verification; and (c) removal of incompatible SNPs for which the effect allele in the exposure dataset did not match either allele in the outcome dataset. Consequently, the selected SNPs were used to assess the potential effect of various types of malocclusion on TMD during the discovery phase. The strength of the IVs was assessed using *F*-statistics, calculated for each SNP as *F* = (β/SE)^2^ using the effect estimates (β) and standard errors (SE) from the exposure GWAS. The mean *F*-statistics ranged from 22.23 for distal bite to 23.62 for scissor bite, with all individual SNP *F*-statistics exceeding 20 (minimum = 20.84), thereby substantially exceeding the Staiger-Stock threshold of 10 for weak instrument bias [13]. Full *F*-statistics for all SNPs are provided in Table 2.

**Table 2.** Summary statistics for the different types of malocclusion.

Analysis	Malocclusion type	Original SNP (*p* < 5 × 10^−6^)	nSNP	LD clumping removed highly linked SNPs	Mean *F*	Min *F*	Max *F*
Forward	Cross bite	73	51	22	23.10	20.87	32.98
Forward	Deep bite	114	90	24	22.46	20.84	28.16
Forward	Deep bite surgery	61	37	24	22.71	20.85	25.54
Forward	Distal bite	59	40	19	22.23	20.85	26.11
Forward	Mesial bite	31	19	12	22.78	21.06	32.91
Forward	Open bite	120	98	22	22.82	20.85	29.14
Forward	Open bite surgery	56	29	27	22.56	20.84	26.83
Forward	Scissor bite	69	45	24	23.62	20.94	28.87
Reverse	TMD	198	130	68	25.97	20.85	31.09

*nSNP: number of SNPs included as instrumental variables in the MR analysis after LD clumping and harmonization; F: F-statistic calculated as F = (β_GWAS_/SE_GWAS_)^2^ from the exposure GWAS; SNP: single-nucleotide polymorphism; TMD: temporomandibular joint disorders; LD: linkage disequilibrium; Min: minimum allowable value; Max: maximum allowable value.*

### 2.3 Statistical analyses

In this study, four MR methods were employed to assess the potential relationship between malocclusion and TMD. The IVW approach constituted the principal analytical technique. Under an ideal scenario in which all IVs are valid and no unbalanced horizontal pleiotropy is present, this method yields the most statistically precise estimate among MR estimators. However, because violation of the no-pleiotropy assumption can introduce non-negligible bias into IVW-derived estimates [14], supplementary analyses were performed using the maximum likelihood, MR-Egger regression [15], weighted median (WM) [16], and penalized weighted median methods.

To verify the reliability of our findings, we conducted a series of sensitivity analyses, including Cochran’s Q test, the MR-Egger intercept test, and the MR-PRESSO global test. The results of Cochran’s Q test were used to assess heterogeneity, with *p* < 0.05 indicating heterogeneity. The MR-Egger intercept test was used to assess horizontal pleiotropy, and MR-PRESSO [17] was applied to detect and correct for outlier SNPs. Furthermore, a leave-one-out sensitivity analysis was performed, whereby each genetic variant was iteratively discarded and the causal estimate was recomputed, to identify whether any single SNP exerted undue leverage on the overall effect.

Statistical significance was set at *p* < 0.00625, based on Bonferroni correction, for primary inferences and *p* < 0.05 for nominal significance, with all tests being two-sided. Results are presented as odds ratios (ORs) with 95% confidence intervals (CIs), representing the change in outcome risk per standard deviation (SD) increase in the exposure. All statistical analyses were performed using R software (v4.3.3), with the TwoSampleMR package (version 0.5.10) and the MRPRESSO algorithm (v1.0).

### 2.4 Reverse Mendelian randomization

In the reverse direction, we investigated whether genetic liability to TMD was causally associated with malocclusion. Because few genome-wide significant SNPs (*p* < 5 × 10^−8^) were available for TMD, a relaxed significance threshold of *p* < 5 × 10^−6^ was used to select TMD-associated SNPs as IVs, consistent with the forward MR approach. A total of 198 TMD-associated SNPs were identified at this threshold. After LD clumping (*r*^2^ < 0.01, 5000-kb window) and harmonization, 130 SNPs were retained as IVs for TMD. The mean *F*-statistic of the TMD-associated SNPs was 22.57 (range: 20.85–31.09), with all individual *F*-statistics exceeding 20.85, thereby substantially exceeding the Staiger-Stock threshold of 10 and indicating negligible weak instrument bias. Only three malocclusion phenotypes, namely distal bite, deep bite, and open bite, were included as outcomes in the reverse analysis, as these showed significant or nominally significant associations in the forward direction.

## 3. Results

### 3.1 Causal association between malocclusion and TMD

MR estimates for various types of malocclusion on TMD in the discovery stage. A total of 19 to 98 SNPs were included as IVs for each malocclusion type after LD clumping and harmonization. The mean *F*-statistic of the exposure-associated SNPs (calculated before clumping) ranged from 22.23 (Distal bite) to 23.62 (Scissor bite), with a minimum individual *F*-statistic of 20.84 across all 582 SNPs, substantially exceeding the Staiger-Stock threshold of 10 and indicating no weak instrument bias (Table 2).

In the forward MR analysis, the statistical power for detecting an OR of 1.1 at the Bonferroni-corrected threshold (α = 0.00625) exceeded 80% for the following phenotypes: Deep bite (83.3%), Distal bite (80.5%), Mesial bite (96.3%), Open bite (98.9%), Open bite Surgery (90.7%), and Scissor bite (81.1%). Power was slightly below 80% for Cross bite (71.3%) and Deep bite Surgery (78.2%). The non-significant association for Scissor bite (OR = 1.02, *p* = 0.542) was adequately powered to exclude moderate causal effects (OR ≥1.1), supporting the interpretation that this null result reflects a genuine absence of association rather than insufficient power. For Cross bite (OR = 1.02, *p* = 0.575), the statistical power was below 80%, and this null result should therefore be interpreted with caution. For Deep bite Surgery, the borderline statistical power (78.2%), together with the significant *p*-value (*p* = 0.0013), which remained significant after Bonferroni correction, suggests that the association is genuine but modest.

For the reverse MR analysis (TMD → malocclusion), the power to detect an OR of 1.1 at α = 0.00625 was 95.0% for Deep bite, whereas it was 78.9% for Distal bite and 78.0% for Open bite. The high statistical power for Deep bite strengthens confidence in the null finding for this phenotype. However, for Distal bite and Open bite, statistical power fell marginally below the conventional 80% threshold; therefore, small causal effects of TMD on these malocclusion types cannot be conclusively excluded.

The MR-PRESSO global test was performed for all eight malocclusion exposures, and no significant outliers were detected for any exposure (all *p* > 0.05): Cross bite (*p* = 0.982, 51 SNPs), Deep bite (*p* = 1.000, 90 SNPs), Deep bite Surgery (*p* = 0.921, 37 SNPs), Distal bite (*p* = 0.999, 40 SNPs), Mesial bite (*p* = 0.985, 19 SNPs), Open bite (*p* = 1.000, 98 SNPs), Open bite Surgery (*p* = 1.000, 29 SNPs), and Scissor bite (*p* = 0.933, 45 SNPs). Consequently, no SNPs were removed as outliers. No heterogeneity was observed between malocclusion and TMD on Cochran’s Q test (Distal bite: IVW Q = 31.330, *p* = 0.599; Deep bite: IVW Q = 85.249, *p* = 0.295; Open bite: IVW Q = 67.623, *p* = 0.715). Leave-one-out analyses demonstrated that the causal estimates were robust across all eight malocclusion types, with no single SNP identified as an influential outlier (all is_outlier values were false). The maximum leave-one-out statistics ranged from 3.42 (Scissor bite) to 7.15 (Deep bite), all of which were below the respective chi-squared thresholds (9.05–12.08). For the four Bonferroni-significant associations specifically, the maximum leave-one-out statistics also remained below the corresponding thresholds: Distal bite (max leaf stat = 4.86, threshold = 10.41), Deep bite (7.15 *vs.* 11.92), Deep bite Surgery (5.17 *vs.* 10.27), and Open bite Surgery (4.18 *vs.* 9.82). The pleiotropy test revealed no significant intercept (Distal bite: Egger intercept = 0.0037, SE = 0.0175, *p* = 0.831; Deep bite: Egger intercept = −0.0084, SE = 0.0119, *p* = 0.480; Open bite: Egger intercept = 0.0249, SE = 0.0135, *p* = 0.069), suggesting no evidence of directional pleiotropy.

Significant evidence indicated that several types of malocclusion were causally associated with TMD. The IVW method (Distal bite: OR = 1.10, 95% CI = 1.05–1.16, *p* = 0.00021; Mesial bite: OR = 1.04, 95% CI = 1.00–1.09, *p* = 0.04095; Cross bite: OR = 1.02, 95% CI = 0.96–1.08, *p* = 0.57519; Scissor bite: OR = 1.02, 95% CI = 0.96–1.07, *p* = 0.54173; Deep bite: OR = 1.14, 95% CI = 1.08–1.20, *p* = 0.00001; Deep bite Surgery: OR = 1.09, 95% CI = 1.03–1.15, *p* = 0.0013; Open bite: OR = 1.05, 95% CI = 1.01–1.09, *p* = 0.0158; Open bite Surgery: OR = 1.11, 95% CI = 1.06–1.16, *p* = 0.00001) revealed that several types of malocclusion were causally related to TMD. However, the MR-Egger results (Distal bite: OR = 1.11, 95% CI = 1.02–1.19, *p* = 0.01546; Mesial bite: OR = 1.01, 95% CI = 0.90–1.13, *p* = 0.85200; Cross bite: OR = 0.97, 95% CI = 0.88–1.07, *p* = 0.58688; Scissor bite: OR = 1.04, 95% CI = 0.96–1.12, *p* = 0.33396; Deep bite: OR = 1.20, 95% CI = 1.07–1.34, *p* = 0.0019; Deep bite Surgery: OR = 1.12, 95% CI = 1.03–1.21, *p* = 0.0100; Open bite: OR = 0.92, 95% CI = 0.86–0.99, *p* = 0.0303; Open bite Surgery: OR = 1.08, 95% CI = 0.99–1.18, *p* = 0.0890) showed significant associations for Distal bite, Deep bite, Deep bite Surgery, and Open bite, although the direction of effect for Open bite differed from that observed in the IVW analysis. The WM and penalized weighted median results for Deep bite Surgery (OR = 1.05, 95% CI = 0.97–1.13, *p* = 0.2178; OR = 1.04, 95% CI = 0.97–1.13, *p* = 0.2703, respectively) showed no significant relationship between Deep bite Surgery and TMD. In summary, the MR analyses of various types of malocclusion on TMD indicated that Distal bite, Deep bite, and Open bite were causally related to TMD (Figs. 2,3).

**Fig. 2.** Estimated association between malocclusion and TMD. (A) Estimated association between malocclusion (distal bite, mesial bite, crossbite, and scissors bite) and TMD. (B) Estimated association between malocclusion (deep bite, open bite, deep bite surgery, and open bite surgery) and TMD. MR: Mendelian randomization; TMD: temporomandibular joint disorders; OR: odds ratio; CI: confidence interval; NA: not applicable.

**Fig. 3.** Sankey plot showing significant associations between malocclusion and TMD. MR: Mendelian randomization; TMD: temporomandibular joint disorders.

Sensitivity analyses, including Cochran’s Q test, the MR-Egger intercept test, and the MR-PRESSO global test, were implemented to assess the reliability of the results above. No heterogeneity was observed between malocclusion and TMD on Cochran’s Q test (Distal bite: IVW Q = 31.330, *p* = 0.599; Deep bite: IVW Q = 85.249, *p* = 0.295; Open bite: IVW Q = 67.623, *p* = 0.715). Additionally, the MR-PRESSO global test yielded consistent results (Distal bite: *p* = 0.476; Deep bite: *p* = 0.345; Open bite: *p* = 0.652), and no outliers were removed. The pleiotropy test revealed no significant intercept (Distal bite: Egger intercept = 0.0037, SE = 0.0175, *p* = 0.831; Deep bite: Egger intercept = −0.0084, SE = 0.0119, *p* = 0.480; Open bite: Egger intercept = 0.0249, SE = 0.0135, *p* = 0.069), suggesting no evidence of directional pleiotropy.

After applying Bonferroni correction (significance threshold = 0.05/8 = 0.00625), Deep bite remained significantly associated with TMD risk (IVW, *p* = 0.0058), whereas Distal bite (MR-Egger, *p* = 0.12368), Open bite (IVW, *p* = 0.1264), and Mesial bite (IVW, *p* = 0.041) did not remain significant at this stricter threshold.

### 3.2 MR estimates for TMD on malocclusion

When 130 TMD-related SNPs were employed as instrumental variables, the reverse MR analysis yielded no support for a causal effect of TMD on malocclusion phenotypes, including Distal bite, Deep bite, and Open bite. The IVW results (Distal bite: OR = 0.97, 95% CI = 0.08–1.18, *p* = 0.7853; Deep bite: OR = 1.11, 95% CI = 0.95–1.29, *p* = 0.1762; Open bite: OR = 1.07, 95% CI = 0.93–1.22, *p* = 0.3461) indicated that TMD was not causally associated with these types of malocclusion. The WM and penalized weighted median results yielded consistent conclusions, showing that TMD was not causally associated with Distal bite, Deep bite, or Open bite (Fig. 4).

**Fig. 4.** Estimated association between TMD and malocclusion. MR: Mendelian randomization; TMD: temporomandibular joint disorders; OR: odds ratio; CI: confidence interval; NA: not applicable.

## 4. Discussion

Utilizing GWAS datasets from the FinnGen and UK Biobank (UKB) consortia, our study employed a bidirectional two-sample MR approach to delineate the causal interplay between malocclusion and TMD. This MR analysis revealed that several types of malocclusion, including distal bite, deep bite, and open bite, were causally associated with TMD, whereas no significant reverse causal effect of TMD on these malocclusion types was observed (Fig. 5). The finding that the causal direction runs from malocclusion to TMD rather than *vice versa* has important clinical implications, underscoring the potential value of timely orthodontic correction as a preventive modality against TMD onset. Consistent with clinical observations, several studies have reported that malocclusion, particularly distal bite, deep bite, and open bite, is associated with TMD [18, 19, 20, 21]. Patients with malocclusion frequently present with concurrent TMD. As malocclusion progresses, inflammation-induced discomfort may result in abnormal occlusal relationships or even occlusal trauma, leading to condylar retrusion, midline deviation, and TMD [22]. It is also plausible that pre-existing joint conditions, such as degenerative changes or disc displacement, could contribute to progressive condylar resorption and the subsequent development of malocclusion, thereby creating a complex bidirectional relationship. However, the absence of significant reverse causal effects in our MR analysis suggests that such joint-to-malocclusion pathways may be less prominent at the genetic level. Hitherto, the question of whether a definitive causal relationship exists between malocclusion and TMD has persisted without resolution.

**Fig. 5.** Summary of the relationship between TMD and malocclusion. TMJ: temporomandibular joint.

The pathological trajectory of TMD is closely tied to condylar architecture, wherein the cartilaginous cap covering the mandibular condyle assumes a pivotal role owing to its marked sensitivity to biomechanical signals. Stress stimulation plays a vital role in regulating bone development and growth [23]. Malocclusion can result in abnormal occlusal forces that increase mechanical compression on the temporomandibular joint, potentially leading to cartilage degradation, subchondral bone remodeling, and eventually TMD [24]. Of particular note, condylar cartilage exhibits heightened vulnerability to modified mechanical loads by virtue of its fibrocartilaginous composition, which possesses inherently constrained regenerative potential relative to hyaline cartilage. Persistent compressive forces arising from occlusal imbalances associated with malocclusion may surpass the reparative threshold of joint tissues, thereby instigating inflammatory cascades and degenerative remodeling [25]. Evidence from biomechanical studies suggests that increased joint loading in malocclusion leads to elevated expression of inflammatory mediators and matrix-degrading enzymes in TMJ cartilage, thereby contributing to the pathogenesis of TMD [26].

However, several limitations should be acknowledged in our study. First, all GWAS repositories were assembled from participants of European heritage, and the allele frequencies and linkage disequilibrium structures underlying our instruments may not transfer seamlessly to populations with different demographic ancestries; replication across East Asian, African, or admixed cohorts is therefore an essential next step. Second, despite our use of summary-level data from nominally separate consortia, partial participant overlap between the exposure and outcome samples cannot be categorically excluded, though any such overlap is anticipated to be modest in magnitude. Third, the selection of genetic instruments relied upon a lenient genome-wide significance threshold (*p* < 5 × 10^−6^) rather than the conventional benchmark, a decision dictated by the need to secure an adequate count of variants. This trade-off between instrument abundance and individual variant strength required us to leverage a comparatively large aggregated sample to enhance statistical power. Fourth, because only GWAS summary statistics were available, we could not perform subgroup analyses based on specific TMD classifications, such as myofascial pain, arthralgia, or disc displacement, according to the DC/TMD criteria. Investigations leveraging patient-level datasets or subtype-specific genome-wide association statistics would materially advance this line of inquiry. Fifth, although MR methodology is expressly constructed to circumvent the confounding that plagues conventional observational designs, it remains theoretically possible that environmental or behavioral covariates may influence the observed associations. For example, environmental factors such as dietary consistency, including hard diet, parafunctional habits such as bruxism, and facial trauma could theoretically influence both the development of malocclusion and the risk of TMD. We attempted to address this issue through multiple sensitivity analyses, including MR-Egger, Weighted Median, and MR-PRESSO, which provided consistent results suggesting that horizontal pleiotropy is unlikely to fully explain our findings. Nevertheless, we cannot completely rule out the possibility that unmeasured environmental or behavioral confounders contribute to the observed associations. Merged genetic–epidemiological frameworks that formally model gene–environment interplay will be essential for unpacking these intricate relationships. Finally, it is important to note that our initial analyses did not correct for multiple comparisons, which may have increased the risk of false-positive findings. After applying Bonferroni correction, only Deep bite remained significantly associated with TMD. Future studies with larger sample sizes and more stringent statistical thresholds are needed to confirm these associations.

## 5. Conclusions

This study provides genetic evidence that the malocclusion–TMD relationship is causal and directional: genetic liability to distal bite, deep bite, and open bite increased TMD risk, with deep bite remaining significant after Bonferroni correction, whereas TMD conferred no causal effect on malocclusion. These estimates remain consistent across complementary analytical estimators, demonstrate robustness against assessments of heterogeneity, pleiotropy and outlier effects, and hold biological plausibility. This can be attributed to the fact that occlusal imbalance associated with malocclusion may impose chronic excessive loading on the temporomandibular joint. Clinically, deep bite may represent modifiable risk factors, highlighting the potential preventive value of timely orthodontic correction. However, our findings are derived from summary-level data of individuals with European ancestry, and do not include stratification by DC/TMD subtypes. Replication studies in diverse populations, as well as subtype-specific and interventional studies, are therefore needed to confirm whether orthodontic treatment can reduce the burden of TMD.
